# Risk factors for back pain in marines; a prospective cohort study

**DOI:** 10.1186/s12891-016-1172-y

**Published:** 2016-07-29

**Authors:** Andreas Monnier, Mats Djupsjöbacka, Helena Larsson, Kjell Norman, Björn O. Äng

**Affiliations:** 1 Department of Neurobiology, Care Sciences and Society, Division of Physiotherapy, Karolinska Institutet, Huddinge, Sweden; 2 Swedish Armed Forces, Regional Medical Service Mälardalen, Berga, Sweden; 3 Centre for Musculoskeletal Research, Department of Occupational and Public Health Sciences, University of Gävle, Gävle, Sweden; 4 Swedish Armed Forces, Headquarters, Medical Services, Stockholm, Sweden; 5 Swedish Armed Forces, 1st Marine Regiment, 2nd Amphibious Battalion, Berga, Sweden; 6 Centre for Clinical Research Dalarna, Falun, Sweden; 7 Karolinska University Hospital, Stockholm, Sweden

**Keywords:** Longitudinal, Military, Movement control, Musculoskeletal disorders, Musculoskeletal injury, Occupational exposure, Prevention, Work ability, Work exposure

## Abstract

**Background:**

It is recognised that back pain (BP) is a debilitating medical problem in the soldier community, which limits operational readiness as well as work ability. As such, identification of risk factors is a necessity for effective preventive actions, but also regarded as important from a safety perspective. The aim of this prospective cohort study was therefore to identify risk factors for back pain and BP limiting work ability in active duty marines within a 6 and 12-month period.

**Methods:**

Demographic characteristics, health-related factors and occupational exposure information, as gathered from questionnaires, as well as clinical test of movement control among 137 Swedish marines were regressed with multivariable logistic regressions, and strength of associations was presented as odds ratio (OR) with 95 % confidence intervals (CI). BP within 6 and 12 months were used as primary outcomes, whereas BP limiting work ability within 6 and 12 months served as secondary outcomes.

**Results:**

Previous BP and tall body height (≥1.86 m) emerged as risk factors for back pain within 6 months (OR 2.99, 95 % CI 1.22–7.30; OR 2.81, 95 % CI 1.16– 6.84, respectively), and 12 months (OR 6.75, 95 % CI 2.30–19.80; 2.75, 95 % CI 1.21–6.29, respectively). Previous BP was also identified as risk factor for BP limiting work ability within 12 months (OR 6.64, 95 % CI 1.78–24.78), and tall body height emerged as a risk within both six (OR 4.30, 95 % CI 1.31–14.13) and 12 months (OR 4.55, 95 % CI 1.53–13.57) from baseline.

**Conclusions:**

Marines with a history of BP are at risk of further BP episodes, which, thus, emphasise the importance of early BP preventive actions. Tall body height also emerged as an important risk which may reflect that personal equipment and work tasks are not adapted for the tallest marines. While this should be considered when introducing new work equipment, further studies are warranted to clarify the underlying mechanism of this association.

**Electronic supplementary material:**

The online version of this article (doi:10.1186/s12891-016-1172-y) contains supplementary material, which is available to authorized users.

## Background

Musculoskeletal disorders (MSDs), i.e. pain, ache or discomforts in the musculoskeletal system, are common in armed forces personnel worldwide [1, 2]. For this occupational group, MSDs constitute not only a major health and economic burden [1, 2], but also limits operational readiness [3] as well as work and training ability [4]. Recent results from our research group [5] indicate MSDs as a widespread problem also in the Swedish Armed Forces (SAF) marines—a highly mobile military unit specialising in operating where water meets land. We found that 46 % of the marines reported back pain (BP) within a 6-month period and two out of five experienced limitations related to their ability to work [5]. For this occupational group, any reduction in work ability is regarded as important from a safety perspective, due to the stern physical demands embedded within the marine’s occupation. As such, prevention of BP is of major importance from both a medical and an operational perspective.

Developing BP is likely to be multifactorial, which include both personal and work-related factors, such as internal tolerance and external load [6, 7]. As such, the identification of both independent and interacting risk factors is a necessity for impacting effective preventive actions. While risk factors for BP in marines are likely to include some of those identified for the general civilian populations, the generalisability of research findings from civilian populations to marines may be uncertain since the latter group is known for working in a specific and very physically-demanding work environment. Further, the unique personal characteristics of marines, i.e. a homogenous young, physically well-trained and primarily male population, may differ from most investigated civilian populations. As a results, research studies have been able to demonstrate that anthropometrical measures and certain work exposures seem to be more specific and relevant risks for BP in military occupations [5, 8–10], compared with the general population [6, 11]. However, to date, the lack of longitudinal studies on such risk factors in non-deployed, active duty marines hinders valid BP preventive measures for this population.

Prior MSDs typically emerge as an important risk for new episodes of MSDs in civilian populations [11] and military personnel alike [12–14]. It is, however, unclear whether such relation is region-specific or reflects general increased vulnerability of the musculoskeletal system, meaning the plausibility of pain in other regions serves as an equally strong predictor of BP as previous BP episodes. Delineating this relation has implications on both primary and secondary preventive actions, with different strategies likely to be adopted for both prevention and treatment, depending on the nature of the underlying risk.

Low performance in standard military physical fitness tests that, typically, measure aerobic capacity, muscle strength or endurance, has previously been suggested as indicating a risk for future BP episodes in conscripts, recruits and deployed personnel [15, 16]. However, for marines in “combat” military occupation specialities where frequent physical exercise is the norm, an additional clinical screening test is warranted. As such, tests of movement control, e.g. the ability to prevent unwanted movements of the back while moving other body parts, are suggested to adequately challenge “weak-links” in the musculoskeletal system relevant to the marines’ occupational tasks. Recent research indicates clinical tests that are supposed to reflect deficiencies in movement control can predict injuries of the back and lower extremities in civilian occupations, including ballet dancers [17] and professional athletes [18]. Research concerning the predictive ability for BP using such clinical tests for physically-demanding occupations, such as marines, is still limited.

In the present study, we aimed to identify individual, clinical, and health- and work-related risk factors for BP in SAF active duty marines within a 6- and 12- month event-window. Secondly, to identify risk factors that may predict BP limiting work ability during the same periods.

## Methods

### Study design

A prospective cohort study design was used on a sample of SAF marine infantry, rangers, and combat craft crews. Baseline measurements were conducted by use of questionnaires and movement-control tests over the course of 1 year, which started in the autumn of 2010 and followed-up after 6 and 12 months. After written and oral information was provided, signed informed consent was obtained from all subjects prior to enrolment. The study was approved by the Regional Medical Research Ethics Committee, Stockholm (2010/728-31/2).

### Study setting and participants

**Fig. 1:**
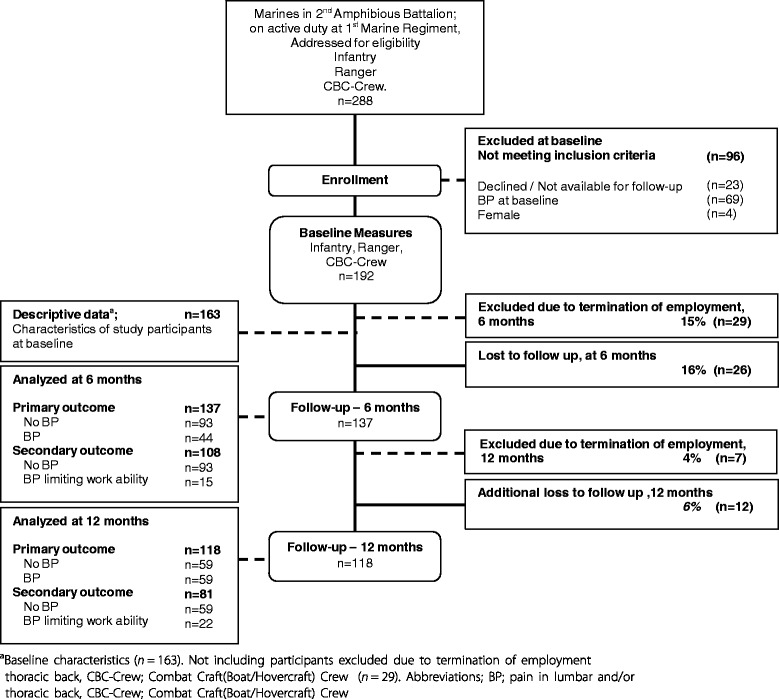
Illustration of recruiting procedure, number of subjects included, excluded, analyzed and lost to follow up

All eligible marines (soldiers and officers) serving as infantry, ranger or combat craft crews at the main marine battalion in Sweden, the 2nd Amphibious Battalion, were invited to participate. To be eligible for enrolment, subjects had to be on active service as a marine, i.e. excluding subjects temporarily posted or undergoing training at the battalion, marines planning to quit SAF during the course of the study, and marines with planned absence during the follow-up period. In addition, female marines (too few for statistical adjustment or stratification) and marines reporting ongoing pain in the back at the time of baseline measurements, defined as ≥1 on numeric pain ratings for lumbar or thoracic back [19], were excluded from the present anlysis. Of the 288 marines initially assessed for eligibility, 192 met the inclusion criteria and provided informed consent to participate, and were subsequently enrolled in the study (Fig. 1). Of these 192 marines, 36 were excluded (29 before the 6 month and an additional seven before the 12-month follow-up) from the analysis (conducted on data succeeding their withdrawal) due to termination of employment.

### Questionnaires

Data on demographics, self-assessed health, health-related function, health-related quality of life, physical training habits, work ability, and work-exposures were collected using four questionnaires. A purpose-built questionnaire was used to capture demographic background information, which included the following: age; body weight; body height; gender and smoking habits. The Musculoskeletal Screening protocol [20] was used to capture physical training habits, specifically, hours per week spent doing strength and aerobic fitness training. The SF-36 Health Survey (IQOLA Short form-36 Standard Swedish version 1.0) addressed self-assessed health and health-related quality of life. Its 36 items sum up to eight scale scores, ranging between 0 and 100, with a higher score indicating a better health outcome, and two norm-based index summaries (with a mean of 50 for the general Swedish population). For the purpose of this study, a questionnaire made up of different items previously used in Swedish public health cohorts was used to capture specific work exposures (type, duration or frequency) [21], work ability [22], recovery/recuperation [23] and days per week with any physical training (>20 min) [21]. It also included ratings on the occurrence of musculoskeletal pain for each anatomical area of the body [24], illustrated by a mannequin [5], as follows: “No pain”; “Pain a couple of days per month or less” and; “Pain a couple of days per week or more” within the last 6 months [5]. For the purpose of this study, musculoskeletal pain was defined as any reported pain experienced in a specific anatomical area, i.e. any of the two pain alternatives. When rating pain within the previous 6 months, subjects were explicitly requested to further state related limitations in work ability as “not limited”, “limited to some extent” or “limited to a large extent”. For the purpose of this study, musculoskeletal pain limiting work ability was defined as any reported pain that limited work ability to at least some extent in that specific anatomical area.

### Clinical tests of movement-control

**Fig. 2:**
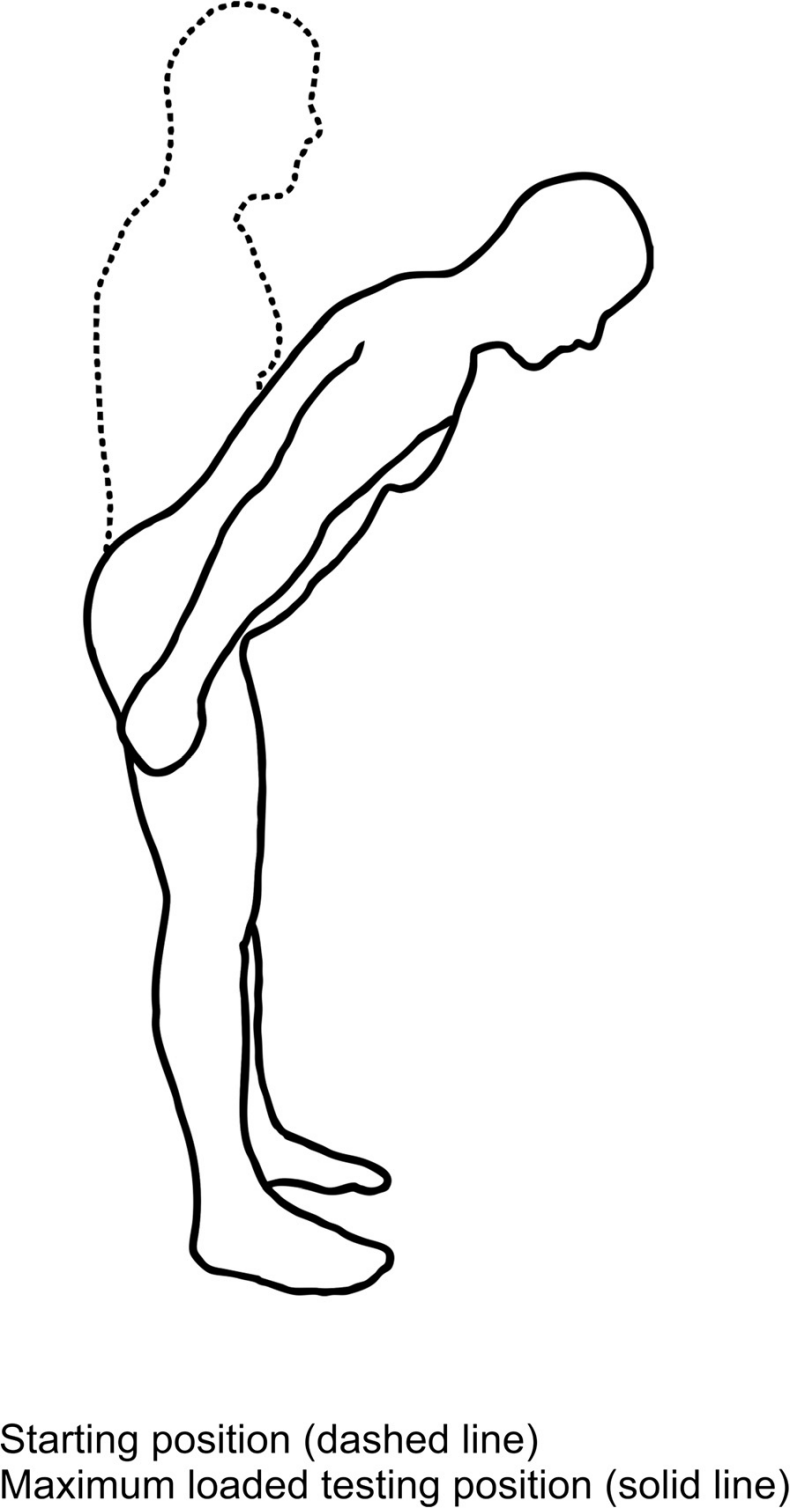
The Standing bow test. (Illustrations adapted from Monnier et al.: Inter- and intra-observer reliability of clinical movement-control tests for marines. BMC Musculoskeletal Disorders 2012 13:263). *Purpose*: To test the ability of preventing flexion of the lumbar spine. *Test movement*: Forward lean by bending the hips to 50° flexion. *Pass criteria*: Maintenance of lumbar spine in a neutral position

**Fig. 3:**
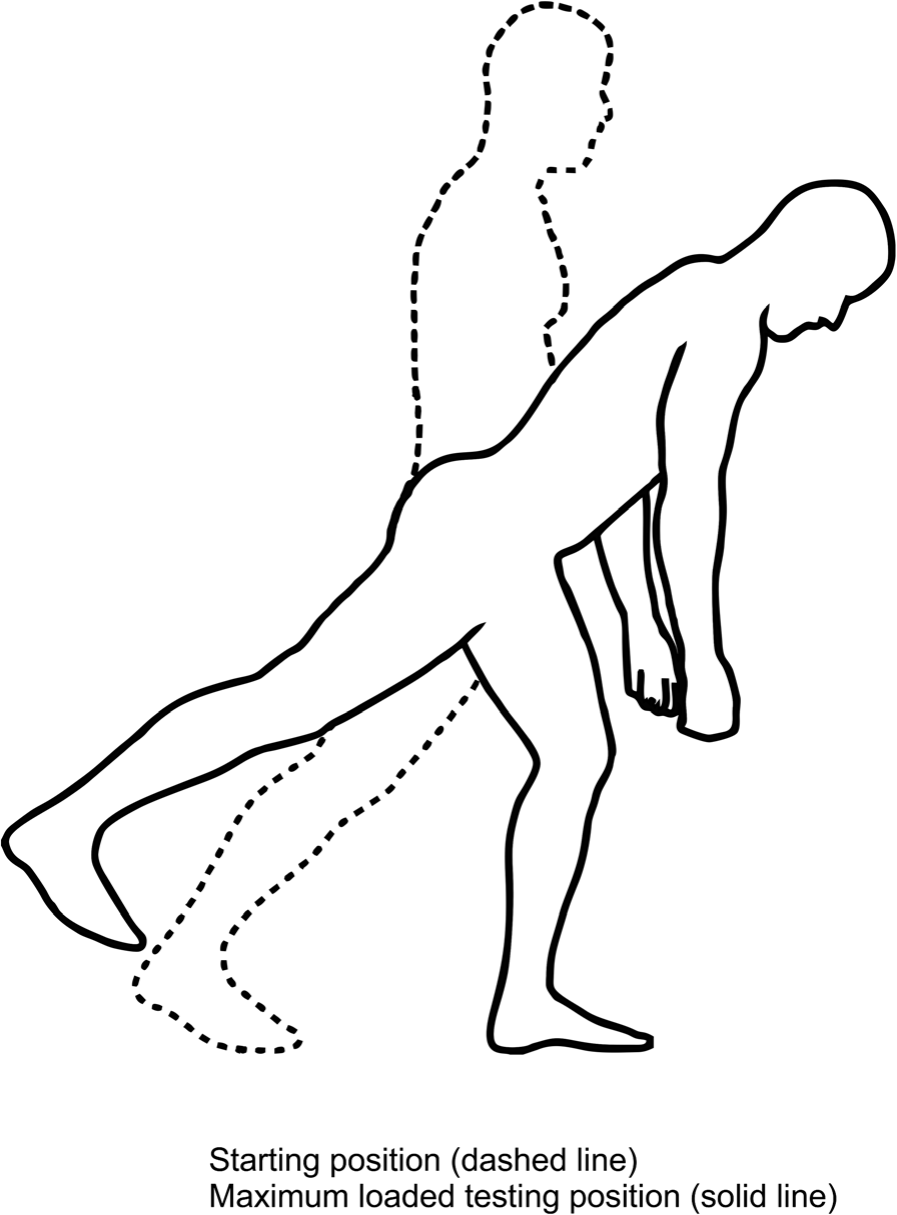
The Single-leg small knee bend + lunge-lean test. (Illustrations adapted from Monnier et al.: Inter- and intra-observer reliability of clinical movement-control tests for marines. BMC Musculoskeletal Disorders 2012 13:263). *Purpose*: To test the ability of preventing extension, flexion, side bend and rotation of the lumbar spine, and control movement of the hips. *Test movement*: Forward lean of the trunk, performed in a single leg knee-bend position, whilst holding the final position for 5 s. *Pass criteria*: The spine in a neutral position without deviations into flexion, extension, side bend or rotation

**Fig. 4:**
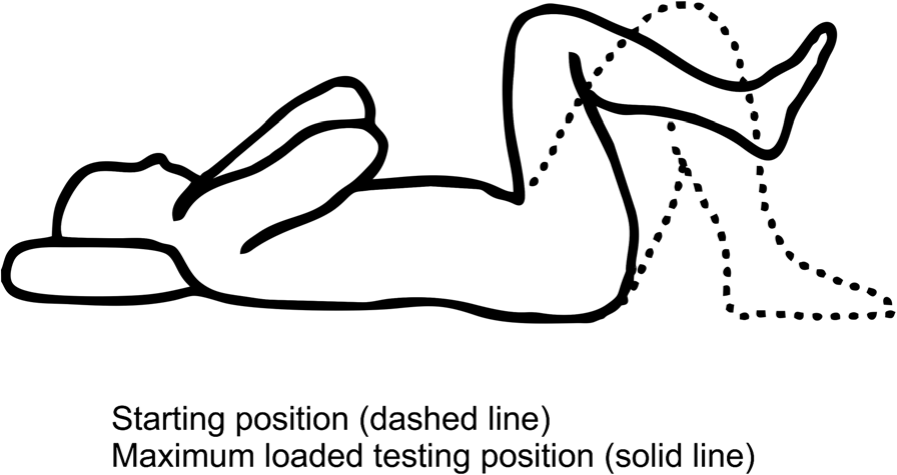
The Double-leg lift-lower test. (Illustrations adapted from Monnier et al.: Inter- and intra-observer reliability of clinical movement-control tests for marines. BMC Musculoskeletal Disorders 2012 13:263). *Purpose*: To test the ability of preventing extension and flexion of the lumbar spine. *Test movement*: A pressure biofeedback unit (Chattanooga Group, Hixon, TN) was initially positioned between the lumbar lordosis and bench surface and inflated to a pressure of 40 mmHg, raising both legs from a supine (crook lying) position to 90° hip flexion and lowering the legs back to the starting position. *Pass Criteria*: <5 mmHg pressure change

**Fig. 5:**
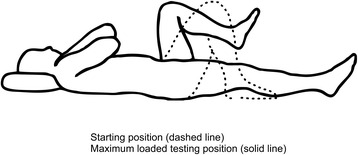
The Double-Leg Lift-Alternate Leg Extension. (Illustrations adapted from Monnier et al.: Inter- and intra-observer reliability of clinical movement-control tests for marines. BMC Musculoskeletal Disorders 2012 13:263). *Purpose*: To test the ability of preventing extension, flexion and rotation of the lumbar spine, as well as leg abduction and lateral rotation. *Test movement*: A pressure biofeedback unit was initially positioned between the lumbar lordosis and bench and inflated to a pressure of 40 mmHg. Then a bilateral leg raise from a supine (crook lying) position was performed to 90° hip flexion, following by a unilateral lowering of the leg to a fully extended position, and then again returning back to 90° hip flexion, repeating with the other leg, and finally bilateral leg lowering back to the crook lying starting position. *Pass Criteria*: <5 mmHg pressure change, with the extending leg not moving away from the midline or turning out

Four active movement control tests [25], described in detail in a previous study [19] and illustrated in Figs. 2, 3, 4, and 5 were used to evaluate the subjects’ ability to control defined movements of the lumbar spine. These tests; *Standing bow* (*SB*), *Single leg small knee bend* + *lunge*-*lean* (*SLKB* + *LL*), *Double leg lift*-*lower* (*DLL*-*L*) and *Double leg lift*-*alternate leg extension* (*DLL*-*ALE*), are routinely used in clinical treatment of the studied population. These tests are also believed, as based on our clinical experience, to adequately challenge weak-links in marines’ musculoskeletal system relevant to their occupational tasks, and have recently been tested by us for reliability and discriminative validity in SAF marines [19]. The testing followed a standardised procedure as described by Monnier et al. [19].

### Outcomes

Our primary outcomes were BP, defined as the presence of any lumbar- and/or thoracic pain, within 6 and 12 months (i.e. any reported BP within any of the two follow-up periods) from baseline, respectively. Our secondary outcomes were BP limiting work ability, defined as the presence of any lumbar- and/or thoracic pain limiting work ability, within 6 and 12 months (i.e. any reported BP limiting work ability to some extent within any of the two follow-up periods) from baseline, respectively. For this outcome, marines with BP limiting work ability were contrasted with marines with no BP; hence, omitting marines reporting pain but not limiting work ability in order to obtain distinct groups (cases vs. references) of marines that experienced BP interfering with their ability to work.

### Independent variables

Twenty-two independent variables (9 individual and health-related, 9 work-related and the results of the 4 clinical movement control tests), as listed below, were analysed as potential risk factors for BP and BP limiting work ability. These were selected based on empirical evidence of risk factors for BP in military and civilian populations.

### Individual and health-related variables

#### Individual characteristics

Based on previous results [5], we postulated that being among the heavier or tallest 1/3 of the sample would be associated with BP. Therefore, body weight and body height were dichotomised as <87 Kg (*reference*) *or* ≥87 Kg, and <1.86 m (*reference*) *or* ≥1.86 m, respectively.

#### Mental and musculoskeletal health

The mental health domain from SF-36, the *MHI*-*5* was dichotomised as <76 or ≥76 (*reference*) out of 100, as previously used in the general population [26]. The presence of *back*, *lower extremity* and *neck*/*shoulder pain* within the preceding 6 month from baseline was dichotomised as yes or no (*reference*) for the respective region.

#### Physical training

A u-shaped relationship with BP was hypothesised for *muscular strength training*, i.e. too little and too much training may both be risks for BP in this group. Consequently, the middle category was used as reference. Due to the lack of valid cut-offs for this population, categories were derived from tertiles as <1.5 h/week, 1.5–4 h/week (*reference*) and >4 h/week. Such a data-driven categorisation was also applied for hours per week with “*aerobic fitness training*”, which yielded three categories with the lowest one considered as reference; <3 h/week (*reference*), 3–6.5 h/week and >6.5 h/week. Additionally, *physical training* <2 days/week, i.e. less than the recommendations for the general population [27] or the physical standards of the SAF, was analysed as a risk factor for BP limiting work ability [5]. This variable was, however, not anlysed for the primary outcome, as to few marines classified as “not training” also experienced BP (yielding a too low expected frequency), which consequently invalidates its use in this type of anlysis.

### Work-related variables

#### Work related exposures

Marines with a “*grade*” of soldier were hypothesised to be at greater risk for BP than marine-officer (*reference*), due to more physically-demanding work tasks and lower socio-economic status, and were dichotomised accordingly. Based on biomechanical exposure characteristics [5], military occupation specialities were categorised as infantry (*reference*), ranger and combat craft crew for the primary outcomes. *The total time working with similar tasks* was categorised as ≤1 year (*reference*), 1–2 years and ≥2 years. Ratings of current *work ability with regard to physical demands of work* and *mental demands of work* were dichotomized as high (*reference*) and moderate (rather good-to-very poor). This was based on the hypothesis that “less-than-optimal” work ability constitutes a risk in this environment. The item “how often the marines felt thoroughly *rested* (*recovered*) *when starting work in the morning*” was dichotomised as always (*reference*) and not always based on the same principle of “less-than-optimal” recovery being regarded as a possible risk.

#### Work-tasks

Proportions of average working day exposed to *vibrating floor*/*seat* and *computer work* were dichotomised as <1/4 of the work day (*reference*) and ≥1/4 of the work day. The cut off for exposure to *vibrating floor*/*seat* was based on the lowest time-limit in military craft potentially associated with BP [28] and for computer work based on previous results from this population, indicating an association with computer work ≥1/4 of the work day and BP limiting work ability [5]. Based on previous results [5], the proportions of average working day exposed to *sitting* was dichotomised as <1/2 of the work day (*reference*) and ≥1/2 of the work day.

### Clinical tests

#### Tests of movement control

Test performance of specific movement directions assessed in the tests, e.g. lumbar flexion and extension, was dichotomised as pass (*reference*) or fail for each test.

### Confounding variables

*Age*, *BMI*, total amount *o*f *weekly physical training*, and *non*-*musculoskeletal co*-*morbidity* were a priori considered possible confounders, defined as a >10 % change in the odds ratio of an independent variable in the adjusted- to crude –model when tested during the analytic process as described below.

### Data management and statistics

#### Missing data and multiple imputation

The main dependent variables, i.e. BP within 6- and 12-months, had 16 and 24 % missing data, respectively, due to subjects missing at follow-up. Additionally, four of the independent variables were missing 12 % of data (clinical tests), one missing 9 % (mental health (MHI-5)), five missing 1–3 % (vibrating floor/seat, aerobic fitness training, physical training, work ability with regard to physical and mental demands of work), and three missing <1 % (prior lower extremity pain, muscular strength training, recovered when starting work in the morning). Analysis of “missing data mechanism”, using a method previously described by Vittinghoff et al. [29], indicated covariate-dependent missing completely at random for one of the dependent variables and missing completely at random for the rest of the outcomes and predictor variables. To reduce risk of bias due to missing data, multiple imputations by chained equations were used to generate 30 imputed datasets [30], with “complete” data on all predictor variables on which pooled statistics were calculated [31]. In the absence of auxiliary variables, i.e. additional variables not included in the analysis but that strongly correlated with variables with missing data, the analysis was restricted to marines having the outcome variables observed in order to not introduce further bias into the model [30, 32]. The analysis method used for the secondary outcomes, as described below, did not support multiple imputed data, i.e. pooled analysis of multiple generated data sets. Therefore, to limit the additive effect of missing data on power, secondary analyses were restricted to complete cases, allowing only <5 % of missing data for independent variables.

#### Statistics

Binary logistic regressions were used to estimate the association of potential risk factors and the primary outcomes, and were reported as odds ratio (OR) with corresponding 95 % CI. To handle potential small sample bias, secondary outcomes were regressed using penalised likelihood (Firth) binary logistic regressions and were reported as OR [33]. As none of the independent variables displayed high risk of co-linearity (Spearman’s *r* < 0.6), independent variables identified, with univariate logistic regressions, to be associated with the dependent variable, at the level of *p* < 0.20, were included in a multivariable logistic regression model. In accordance with the purposeful selection process described by Hosmer, Lemeshow and Sturdivant [34], an iterative process followed by deleting non-significant variables, *p* > 0.05, or non-confounding variables and then refitting and evaluating the model until yielding a final model containing only significant (*p* < 0.05) independent variables, identified plausible confounders (also including previously excluded variables refitted and evaluated for a confounding effect) and interactions. Interactions, between independent variables in the final model, were included in the final model if significant at the level of *p* < 0.05. Also, for the primary outcomes, interactions between specific variables in the final model and omitted variables were addressed if plausible from a clinical perspective (e.g. potential interaction between pain in more than one body area). All final models were deemed to have sufficient power based on the events-per-variable ratio [35], and showed good fit as determined by methods described by Hosmer, Lemeshow and Sturdivant [34] and Vittinghoff [29]. Analysis was performed using IBM SPSS Statistics for Windows (version 22.0; Armonk, NY) and STATA Statistical software (version 13.1; College Station, TX).

## Results

Table 1 provides participant characteristics at baseline. The cohort comprised primarily of marines with the grade of soldier (78 %) and having an occupational specialty as marine infantry (66 %). Twenty-six marines were lost to follow up at 6 months and, additionally, 12 more were lost to follow up at the 12-month interval. Compared with the analysed sample, these marines were older with a mean (Sd) of 26.0 (5.9) years, but did not differ with regard to prior MSDs. A separate analysis of the 36 marines excluded due to quitting the SAF, indicated younger marines, non-officers and marines reporting non-musculoskeletal diagnoses, such as cancer, cardiovascular or respiratory disease, to be more likely to terminate their employment in SAF during the course of the study. Nonetheless, these marines did not differ from those analysed with regard to prior MSDs.

**Table 1:** Demographic data, self-rated general health and health-related quality of life at baseline^a^

	Mean	SD
Age (years)	23.6	4.3
Body weight (kg)	83.1	10.1
Body height (m)	1.83	0.06
Body mass index (kg/m^2^)	24.9	2.4
	%	95 % CI
Smoker		
No	90.1	84.7–93.9
Occasionally	9.8	6.1–15.4
Yes	0	
Non musculoskeletal co-morbidity^b^	4.3	2.1–8.6
General Health		
Good	95.6	91.3–97.9
Less than good	4.4	2.1–8.8
	Mean	SD
Health-related quality of life		
Physical component summary^c^	54.4	4.6
Mental component summary^c^	53.0	6.0
	%	95 % CI
Military occupational function		
Infantry (Assault)	65.6	58.1–72.5
Ranger	17.8	12.7–24.4
CBC-Crew^d^	16.6	11.6–23.0
Grade		
Officer	22.1	16.4–29.1
Soldier	77.9	70.9–83.6

Reported with mean and standard deviation (SD) or percent and corresponding 95 % confidence interval (95 % CI)

^a^Baseline characteristics (*n* = 163). Not including complete withdrawals (*n* = 29)

^b^Non-musculoskeletal diagnosis, such as, cancer, cardiovascular or respiratory disease

^c^Norm-based score (mean of 50 for the general Swedish population)

^d^CBC-Crew; Combat Craft(Boat/Hovercraft) Crew

In total, 137 (44 BP cases) marines were included in the regression analyses for BP within 6 months and 118 (59 BP cases) marines for BP within 12 months. From the univariate regressions, one individual, two health-related, three work-related risk factors and the results of one movement control test were associated, at *p* < 0.20, with BP within 6 months. For BP within 12 months, two individual, three health-related, four work-related risk factors and the results of a second movement control test emerged associated (Table 2). Variables not associated at *p* <0.20 in the univariate analysis are presented in the Additional file 1: Table S1.

**Table 2:** Univariate regression analyses: variables associated with back pain within 6 and 12 months of baseline

	Back pain within 6 months						Back pain within 12 months					
	n^a^	Cases^a^	(%)	OR	95 % CI	*p*-value	n^a^	Cases^a^	(%)	OR	95 % CI	*p*-value
Individual factors												
*Body weight* (*Kg*)												
< 87	.	.	.	.	.	.	82	37	(45)	1.00	*Reference*	.
≥ 87	.	.	.	.	.	.	36	22	(61)	1.91	0.86–4.25	0.112
*Body height* (*m*)												
< 1.86	90	23	(26)	1.00	*Reference*	.	74	30	(41)	1.00	*Reference*	.
≥ 1.86	47	21	(45)	2.35	1.12–4.96	0.024	44	29	(66)	2.84	1.30–6.17	0.009
Health-related factors												
*Previous back pain* ^*b*^												
No	75.6	17	(22)	1.00	*Reference*	.	90	36	(40)	1.00	*Reference*	.
Yes	61.4	27	(44)	3.60	1.59–8.14	0.002	28	23	(82)	6.90	2.40–19.82	<0.001
*Previous lower extremity pain* ^*b*^												
No	104	26	(25)	1.00	*Reference*	.	66.6	27	(41)	1.00	*Reference*	.
Yes	33	18	(55)	2.71	1.29–5.68	0.008	51.4	32	(62)	2.43	1.45–5.14	0.021
*Previous neck*/*shoulder pain* ^*b*^												
No	.	.	.	.	.	.	83	38	(46)	1.00	*Reference*	.
Yes	.	.	.	.	.	.	35	21	(60)	1.78	0.80–3.96	0.160
Work-related factors												
*Military occupational function*												
Infantry (Assault)	93	26	(28)	1.00	*Reference*	.	81	36	(44)	1.00	*Reference*	.
Ranger	24	7	(29)	1.06	0.39–2.86	0.907	20	11	(55)	1.52	0.57–4.09	0.399
CBC-Crew^c^	20	11	(55)	3.15	1.17–8.48	0.023	17	12	(71)	3.00	0.97–9.30	0.057
*Sitting work*												
< 1/2 work day	111	31	(28)	1.00	*Reference*	.	93	42	(45)	1.00	*Reference*	.
≥ 1/2 work day	26	13	(50)	2.58	1.08–6.18	0.033	25	17	(68)	2.58	1.01–6.57	0.047
*Computer work*												
< 1/4 work day	112	33	(29)	1.00	*Reference*	.	93	43	(46)	1.00	*Reference*	.
≥ 1/4 work day	25	11	(44)	1.88	0.77–4.57	0.163	25	16	(64)	2.07	0.83–5.15	0.119
*Vibrating floor*/*seat*												
< 1/4 work day	.	.	.	.	.	.	86.8	39.8	(46)	1.00	*Reference*	.
≥ 1/4 work day	.	.	.	.	.	.	31.2	19.2	(62)	1.89	0.82–4.38	0.135
Clinical test of movement control												
*Standing bow*												
Pass	.	.	.	.	.	.	76.2	42.2	(55)	1.00	*Reference*	.
Fail	.	.	.	.	.	.	41.8	16.8	(40)	0.54	0.24–1.20	0.129
*Double leg lift*-*lower*												
Pass	76.6	30.1	(39)	1.00	*Reference*	.	.	.	.	.	.	.
Fail	60.4	13.9	(23)	0.46	0.21–1.01	0.054	.	.	.	.	.	.

odds ratio (OR) with corresponding 95 % confidence interval (95 % CI) and significance level (*p*-value) for variables associated at *p* <0.20 with back pain within 6 and 12 months of baseline

^a^Based on pooling of 30 imputed datasets

^b^Pain within 6 months prior to baseline

^c^CBC-Crew; Combat Craft(Boat/Hovercraft) Crew

^.^Category not applicable

Table 3 presents the results of the final multiple logistic regression models for BP within 6 and 12 months. Following the purposeful selection process, *Previous BP* and *tall body height* (≥*1.86 m*) emerged as independent risk factors for BP within both 6 and 12 months. For BP within 6 months, *prior lower extremity pain* and *sitting work on average* ≥*1*/*2 of the working day* were also identified as independent risks, while failure on the *DLL*-*L test* showed a protective effect on BP. No confounding variables emerged, neither were any significant interactions (between any of the variables in the final models, between the pain variables and pain in different body regions, or between body height and military occupation specialty) observed in the final models of BP at either 6 or 12 months; therefore, none were included in the final models.

**Table 3:** Multiple regression analyses: odds ratio for back pain within 6 and 12 months of baseline

	Back pain within 6 months			Back pain within 12 months		
	OR	95 % CI	*p*-value	OR	95 % CI	*p*-value
*Body height* (*m*)						
< 1.86	1.00	*Reference*	.	1.00	*Reference*	.
≥ 1.86	2.81	1.16–6.84	0.022	2.75	1.21–6.29	0.016
*Previous back pain* ^a^						
No	1.00	*Reference*	.	1.00	*Reference*	.
Yes	2.99	1.22–7.30	0.016	6.75	2.30–19.80	0.001
*Previous lower extremity pain* ^*a*^						
No	1.00	*Reference*	.	.	.	.
Yes	2.32	1.02–5.24	0.044	.	.	.
*Sitting work*						
< 1/2 work day	1.00	*Reference*	.	.	.	.
≥ 1/2 work day	2.78	1.06–7.32	0.038	.	.	.
*Double leg lift*-*lower*						
Pass	1.00	*Reference*	.	.	.	.
Fail	0.32	0.12–0.82	0.018	.	.	.

odds ratio (OR) with corresponding 95 % confidence interval (95 % CI) and significance level (*p*-value) for back (lumbar and thoracic) pain within 6 and 12 months of baseline

^a^Pain within 6 months prior to baseline

^.^Category not applicable

After excluding marines rating pain in the back that did not limit work ability, 108 (15 BP cases) and 81 (22 BP cases) marines, at 6 and 12 months respectively, were included in the regression analyses of BP limiting work ability. Additional file 1: Table S2 present the results of the initial univariate logistic regression analyses for BP limiting work ability. Following the purposeful selection process, *tall body height* (≥*1.86 m*) (OR 4.30, 95 % CI 1.31–14.13) and service as *combat craft crew* (OR 5.87, 95 % CI 1.58–21.81), adjusted for confounding effect of *previous BP*, were identified as independent risk factors for BP limiting work ability within 6 months. *Tall body height* (OR 4.55, 95 % CI 1.53–13.57) and *previous BP* (OR 6.64, 95 % CI 1.78–24.78) emerged as risk factors for BP limiting work ability within 12 months. Final unadjusted and adjusted models for back pain limiting work ability are presented in Additional file 1: Table S3 and S4.

## Discussion

This first prospective cohort study of MSDs in Swedish Armed Forces (SAF) marines identified significant individual, health- and work- related risk factors for BP within a 6- and 12- month event window. Previous experienced BP pain episodes and being among the taller marines emerged as consistent risks for further BP episodes, while previous pain in the lower extremity and occupational sitting predicted BP only in the shorter perspective.

The present cohort can be regarded as an adequately representative military marine sample since the demographic characteristics were similar to other military cohorts of light mobile infantry units [4, 36]. The external validity, hence, extends primarily to marines and possible similar military units on active national duty. Our definition of pain as “any self-reported pain episode” might be considered too inclusive, possibly including very mild BP that may not interfere with work or other activities. Still, we elected to use this definition since our clinical experience with this type of soldier, supported by studies on similar types of military populations [37], suggests that they are prone to underestimating pain intensity. While also including marines with a history of BP episodes in the present analysis, one could argue that this attenuates the temporal relationship with the exposure. However, such an approach enabled modelling of previous pain episodes as a predictor of new episodes. As a sensitivity analysis of the adequacy of our adjustments, we repeated the analysis of the final models including only marines pain-free 6 months prior to baseline. Despite this, the main results did not show any substantial change, which indicated sufficient adjustment in the present models. It is important, however, to underline that this analysis does not differentiate between first ever back pain and recurrent back pain episodes, potentially due to different risk factors. To reduce the risk of type I error, we included potential predictors only based on empirical evidence and a priori considered biological plausibility and fitted only models supported by our sample size [35]. Even so, borderline significant predictors should be interpreted with caution due to multiple hypotheses testing during the model building process.

As expected, the strongest risk for future BP was a history of previous BP. While in line with studies from the general working population [38, 39] and athletes alike [40], it remained significant in the present study despite adjusting for pain in other body regions. This is important from a clinical perspective, as it is likely more effective to design preventative actions against deficits in specific regions, rather than for the whole body. Its significance in this context further emphasises the important role of previous pain episodes in the aetiology of back pain, and shows that it is valid also in specific populations—like the SAF marines. In addition to a history of BP, pain in the lower extremities (hip, knee or foot) also predicted BP within 6 months. We believe this to reflect marines not yet fully recovered from a previous pain episode of the lower extremities, leaving deficiencies in strength, joint stability or proprioception. In theory, such insufficiencies could increase the demands on the spinal system, and consequently predispose marines with such profiles to future BP episodes, as previously seen in civilian athletes [41]. Such regional-interdependence was, however, not identified for previous pain in the neck/shoulder region or when analysing BP within a 12-month event window. Neither could we identify any alteration of the risk for future BP in the presence of a history of pain in more than one anatomical region, nor in combination with any other identified independent risk factor. While physiological distress and lifestyle factors have been shown to predict new low back pain (LBP) episodes [38, 39], mental health (as measured with MHI-5) or physical exercise habits were not independently—nor in combination with a history of BP—associated with BP in the present study. Such lack of interaction with other risks provides little insight into the specific mechanisms behind recurrent BP. Even so, the present result highlights both the necessity of early primary prevention of BP and the need for future development of effective secondary preventive actions as a way of stopping single BP episodes becoming a recurrent problem or reducing work ability.

In the present study, marines with a body height over 1.86 m consistently emerged as being at greater risk for BP and BP limiting work ability compared to shorter marines. While tall body height has been identified as a risk factor for BP in some civilian studies [42, 43], others have failed to confirm this association [44] or have shown inconsistent results [39, 45]. One potential explanation for the present associations might be that taller marines handle heavy loads in biomechanically unfavourable situations, which consequently increases internal loads of the back due to increased leverages. One could also suspect that if certain equipment, such as, backpacks and body armour, are not appropriately adjustable for the tallest marines, load-reducing functions like waist belts might not work optimally. Tall body height could thus constitute a risk factor when in conjunction with high physical work demands, a common feature for all marines in the present study but possibly not present, or sufficiently controlled for, in other occupational cohorts. Such interaction with generic occupational exposures could potentially also explain why long periods in a seated posture in this cohort, in contrast with the general populations [46], was identified as a weak-to-moderate risk factor for BP. This is further supported by the findings that occupational sitting has shown a preventive effect on LBP in studies of the general populations [46], but to be a risk when sitting for more than half a day whilst simultaneously being exposed to whole body vibrations or ergonomically unfavourable postures [47]. Hence, such co-exposures could potentially explain why marine combat craft-crews in the present study had a fivefold increase in odds of experiencing BP that limited work ability, compared to rangers and infantry marines.

In contrast with our expectations, it was notable that failing the DLL-L movement control test, i.e. not having the ability to control “low load” lumbar extension/flexion in the test situation, reduced the risk of experiencing BP. For this population this could indicate that movement control necessary to pass the test is achieved by means of co-contraction of trunk muscles, constituting an undue load on the spinal structures [48]. An alternative explanation could be that subjects with good movement control under low load adapt the same, potentially ineffective, strategy during more demanding situations. In contrast, subjects failing to control low-load might adapt a different strategy that is more suitable to that load; hence protecting them from experiencing BP. Admittedly, the dichotomisation of performance on specific movement directions into an overall pass/fail for each test could possibly dilute the present results. As such, further examination of specific movement direction for these tests might still be warranted, while the use of the tests in the present form should be questioned for preventive screening of back pain in marines.

## Conclusion

Previous experienced BP is a strong and consistent risk for further back pain episodes in this occupational group. This underlines the importance of prevention of first ever BP episodes as well as effective secondary preventive strategies for BP in marines. Tall body height also emerged as an important risk and may be due to equipment and work-tasks not adapted for the tallest marines. While this should be considered when introducing new work equipment in this population, the underlying mechanism needs to be further studied.

## Abbreviations

BP, back pain; CI, confidence intervals; DLL-L, double leg lift-lower; DLL-ALE, double leg lift-alternate leg extension; LBP, low back pain; MHI-5, the mental health domain from SF-36; MSDs, musculoskeletal disorders; OR, odds ratio; SAF, Swedish Armed Forces; SB, standing bow; SLKB + LL, single leg small knee bend + lunge-lean

## Additional file

Additional file 1: Table S1. Univariate regression analyses: odds ratio and 95 % CI for variables not associated (*p* >0.20) with back pain at 6 and 12 months follow up. **Table S2.** Univariate regression analyses: odds ratio and 95 % CI for back pain limiting work ability at 6 and 12 months follow up. **Table S3.** Multiple regression analyses: final unadjusted and adjusted odds ratio for back pain limiting work ability within 6 months from baseline. **Table S4.** Multiple regression analyses: final odds ratio for back pain limiting work ability within 12 months from baseline. (DOCX 36 kb)
