# Supplementary material for: Risk factors for back pain in marines; a prospective cohort study
Source: BMC Musculoskelet Disord. 2016 Jul 29;17:319. doi: 10.1186/s12891-016-1172-y (PMC4966738; doi:10.1186/s12891-016-1172-y)
Supplement: Additional file 1: Table S1. — Univariate regression analyses: odds ratio and 95 % CI for variables not associated (p >0.20) with back pain at 6 and 12 months follow up. Table S2. Univariate regression analyses: odds ratio and 95 % CI for back pain limiting work ability at 6 and 12 months follow up. Table S3. Multiple regression analyses: final unadjusted and adjusted odds ratio for back pain limiting work ability within 6 months from baseline. Table S4. Multiple regression analyses: final odds ratio for back pain limiting work ability within 12 months from baseline. (DOCX 36 kb) [file 12891_2016_1172_MOESM1_ESM.docx]

**Additional file**

Table S1. Univariate regression analyses: odds ratio (OR) and 95%CI for variables not associated (p >0.20) with back pain at six and 12 months follow up

|  | | Back pain within 6 mo | | | | | |  |  | | Back pain within 12 mo | | | | |
| --- | --- | --- | --- | --- | --- | --- | --- | --- | --- | --- | --- | --- | --- | --- | --- |
|  |  | n ^a^ | Cases ^a^ | (%) | OR^†^ | 95% CI | p-value |  | n ^a^ | Cases ^a^ | | (%) | OR^†^ | 95% CI | p-value |
| **Individual factors** | |  |  |  |  |  |  |  |  | |  |  |  |  |  |
| *Body weight (Kg)* | | 137 |  |  |  |  |  |  |  | |  |  |  |  |  |
|  | < 87 | 95 | 25 | (23) | 1.00 | Reference | . |  |  | |  |  |  |  |  |
|  | ≥ 87 | 42 | 16 | (38) | 1.47 | 0.69-3.16 | 0.320 |  |  | |  |  |  |  |  |
| **Health-related factors** | |  | |  |  |  |  |  |  | |  |  |  |  |  |
| *Muscular strength training* | | | |  |  |  |  |  |  | |  |  |  |  |  |
|  | < 1.5 hours/week | 40.3 | 12 | (30) | 0.82 | 0.35-1.93 | 0.649 |  | 38.3 | | 20 | (52) | 0.96 | 0.42-2.22 | 0.923 |
|  | 1.5-4.0 hours/week | 61.5 | 21 | (34) | 1.00 | Reference | . |  | 52.5 | | 28 | (53) | 1.00 | Reference | . |
|  | >4.0 hours /week | 35.2 | 11 | (31) | 0.88 | 0.36-2.13 | 0.773 |  | 27.2 | | 11 | (40) | 0.60 | 0.23-1.53 | 0.280 |
| *Aerobic fitness training* | | | |  |  |  |  |  |  | |  |  |  |  |  |
|  | < 3 hours/week | 51.4 | 16.0 | (31) | 1.00 | Reference | . |  | 42.8 | | 24.5 | (57) | 1.00 | Reference | . |
|  | 3-6.5 hours/week | 42.4 | 13.7 | (32) | 1.06 | 0.43-2.59 | 0.900 |  | 34.7 | | 15.4 | (44) | 0.59 | 0.24-1.48 | 0.261 |
|  | >6.5 hours/week | 43.1 | 14.3 | (33) | 1.11 | 0.46-2.66 | 0.822 |  | 40.5 | | 19.1 | (47) | 0.67 | 0.28-1.59 | 0.363 |
| *MHI-5* | | | |  |  |  |  |  |  | |  |  |  |  |  |
|  | < 76 | 14.4 | 4.4 | (31) | 0.91 | 0.27-3.10 | 0.88 |  | 12.7 | | 8.5 | (67) | 2.22 | 0.63-7.84 | 0.217 |
|  | ≥ 76 | 122.6 | 39.6 | (32) | 1.00 | Reference | . |  | 105.3 | | 50.5 | (48) | 1.00 | Reference | . |
| *Prior neck/shoulder pain ^b^* | | | |  |  |  |  |  |  | |  |  |  |  |  |
|  | No | 99 | 31 | (31) | 1.00 | Reference | . |  |  | |  |  |  |  |  |
|  | Yes | 38 | 13 | (34) | 1.14 | 0.52-2.52 | 0.75 |  |  | |  |  |  |  |  |
| **Work-related factors** | |  |  |  |  |  |  |  |  | |  |  |  |  |  |
| *Grade* | | | |  |  |  |  |  |  | |  |  |  |  |  |
|  | Officer | 27 | 10 | (37) | 1.00 | Reference | . |  | 28 | | 16 | (57) | 1.00 | Reference | . |
|  | Soldier | 110 | 34 | (31) | 0.76 | 0.32-1.83 | 0.542 |  | 90 | | 43 | (48) | 0.69 | 0.29-1.61 | 0.388 |

| *Total time working with similar tasks* | | | |  |  |  |  |  |  |  |  |  |  |  |
| --- | --- | --- | --- | --- | --- | --- | --- | --- | --- | --- | --- | --- | --- | --- |
|  | ≤ 12 months | 101 | 34 | (34) | 1.00 | Reference | . |  | 85 | 43 | (51) | 1.00 | Reference | . |
|  | 13-24 months | 16 | 4 | (25) | 0.68 | 0.20-2.19 | 0.494 |  | 16 | 7 | (44) | 0.76 | 0.26-2.28 | 0.616 |
|  | ≥ 25 months | 20 | 6 | (30) | 0.85 | 0.30-2.39 | 0.751 |  | 17 | 9 | (53) | 1.10 | 0.39-3.12 | 0.859 |
| *Current Work ability, Physical ^c^* | | | |  |  |  |  |  |  |  |  |  |  |  |
|  | High | 46.3 | 13 | (28) | 1.00 | Reference | . |  | 38.1 | 17 | (45) | 1.00 | Reference | . |
|  | Moderate | 90.7 | 31 | (34) | 1.33 | 0.62-2.89 | 0.471 |  | 79.9 | 42 | (52) | 1.38 | 0.63-2.99 | 0.421 |
| *Current Work ability, Mental ^c^* | | | |  |  |  |  |  |  |  |  |  |  |  |
|  | High | 102.3 | 33 | (32) | 1.00 | Reference | . |  | 87.5 | 42 | (48) | 1.00 | Reference | . |
|  | Moderate | 34.7 | 11 | (32) | 0.98 | 0.46-2.24 | 0.952 |  | 30.5 | 17 | (56) | 1.37 | 0.59-3.14 | 0.465 |
| *Recovered when starting work* | | | |  |  |  |  |  |  |  |  |  |  |  |
|  | Always | 21.2 | 6 | (28) | 1.00 | Reference | . |  | 20 | 11 | (55) | 1.00 | Reference | . |
|  | Not always | 115.8 | 38 | (33) | 1.23 | 0.44-3.43 | 0.69 |  | 98 | 48 | (49) | 0.79 | 0.30-2.06 | 0.624 |
| *Vibrating floor/seat* | |  | |  |  |  |  |  |  |  |  |  |  |  |
|  | < 1/4 work day | 98.3 | 28.8 | (29) | 1.00 | Reference | . |  |  |  |  |  |  |  |
|  | ≥ 1/4 work day | 38.7 | 15.2 | (39) | 1.57 | 0.72-3.45 | 0.259 |  |  |  |  |  |  |  |
| **Movement control tests** | | | |  |  |  |  |  |  |  |  |  |  |  |
| *Standing bow* | | | |  |  |  |  |  |  |  |  |  |  |  |
|  | Pass | 87.5 | 29 | (33) | 1.00 | Reference | . |  |  |  |  |  |  |  |
|  | Fail | 49.5 | 15 | (30) | 0.87 | 0.40-1.92 | 0.735 |  |  |  |  |  |  |  |
| *Single leg lunge & lean + small knee bend* | | | |  |  |  |  |  |  |  |  |  |  |  |
|  | Pass | 53.4 | 17.5 | (33) | 1.00 | Reference | . |  | 45.3 | 22.1 | (49) | 1.00 | Reference | . |
|  | Fail | 83.6 | 26.5 | (32) | 0.96 | 0.45-2.06 | 0.909 |  | 72.7 | 36.9 | (51) | 1.08 | 0.49-2.38 | 0.840 |
| *Double leg lift-lower* | | | |  |  |  |  |  |  |  |  |  |  |  |
|  | Pass |  |  |  |  |  |  |  | 66.4 | 33.9 | (51) | 1.00 | Reference | . |
|  | Fail |  |  |  |  |  |  |  | 51.6 | 25.1 | (49) | 0.91 | 0.42-1.20 | 0.813 |
| *Double leg lift-alternate leg extension* | | | |  |  |  |  |  |  |  |  |  |  |  |
|  | Pass | 58.1 | 17.9 | (31) | 1.00 | Reference | . |  | 45.8 | 21 | (46) | 1.00 | Reference | . |
|  | Fail | 78.9 | 26.1 | (33) | 1.11 | 0.51-2.39 | 0.797 |  | 72.2 | 38 | (53) | 1.312 | 0.61-2.84 | 0.491 |

^a^ Based on pooled results.

^b^ Pain within 6 month prior to baseline.

^c^Current Work ability in regards to demands of work.

Table S2 Univariate regression analyses: odds ratio (OR)^a^ and 95% CI^b^ for back pain limiting work ability at six and 12 months follow up.

|  | | | | Back pain limiting work ability, within 6 mo | | | | |  | Back pain limiting work ability, within 12 mo | | |
| --- | --- | --- | --- | --- | --- | --- | --- | --- | --- | --- | --- | --- |
|  | | | | OR ^a^ | | | 95% CI | p-value |  | OR ^a^ | 95% CI | p-value |
| **Individual factors** | | | |  | | |  |  |  |  |  |  |
| *Body weight (Kg)* | | | |  | | |  |  |  |  |  |  |
|  | < 87 | | | 1.00 | | | *Reference* |  |  |  | *Reference* |  |
|  | ≥ 87 | | | 2.25 | | | 0.76-6.62 | 0.142 |  | 2.21 | 0.78-6.12 | 0.128 |
| *Body height (m)* | | | |  | | |  |  |  |  |  |  |
|  | < 1.86 | | | 1.00 | | | *Reference* |  |  | 1.0 | *Reference* |  |
|  | ≥ 1.86 | | | 4.86 | | | 1.58-15.00 | 0.006 |  | 4.08 | 14.48-11.22 | 0.006 |
| **Health-related factors** | | | |  | | |  |  |  |  |  |  |
| *Physical training* | | | |  | | |  |  |  |  |  |  |
|  | *≥ 2 days/*week | | | 1.00 | | | *Reference* |  |  | 1.00 | *Reference* |  |
|  | *<2 days/*week | | | 6.3 | | | 1.57-25.26 | 0.009 |  | 3.88 | 1.00-15.05 | 0.050 |
| *Muscular strength training* | | | | | | | |  |  |  |  |  |
|  | < 1.5 hours/week | | | 1.23 | | | 0.39-3.91 | 0.724 |  | 1.32 | 0.45-3.91 | 0.611 |
|  | 1.5-4.0 hours/week | | | 1.00 | | | *Reference* |  |  | 1.00 | *Reference* |  |
|  | >4.0 hours /week | | | 0.55 | | | 0.12-2.511 | 0.441 |  | 0.70 | 0.19-2.54 | 0.591 |
| *Aerobic fitness training* | | | | | | | |  |  |  |  |  |
|  | < 3 hours/week | | | 1.00 | | | *Reference* |  |  | 1.00 | *Reference* |  |
|  | 3-6.5 hours/week | | | 0.75 | | | 0.21-2.66 | 0.653 |  | 0.59 | 0.18-1.88 | 0.371 |
|  | >6.5 hours/week | | | 0.21 | | | 0.21-0.266 | 0.653 |  | 0.53 | 0.17-1.70 | 0.287 |
| *MHI-5* | | | | | | | |  |  |  |  |  |
|  | < 76 | | | 2.72 | | | 0.68-10.86 | 0.157 |  | 2.33 | 0.52-10.4 | 0.268 |
|  | ≥ 76 | | | 1.00 | | |  |  |  | 1.00 |  |  |
| *Prior back pain^b^* | | | | | | | |  |  |  |  |  |
|  | No | | | 1.00 | | |  |  |  | 1.00 |  |  |
|  | Yes | | | 3.47 | | | 1.11-10.80 | 0.032 |  | 5.81 | 1.72-19.65 | 0.005 |
| *Prior lower extremity pain^b^* | | | | | | | |  |  |  |  |  |
|  | No | | | 1.00 | | |  |  |  | 1.00 |  |  |
|  | Yes | | | 4.33 | | | 1.35-13.94 | 0.014 |  | 3.46 | 1.26-9.45 | 0.016 |
| *Prior neck/shoulder pain^b^* | | | | | | | |  |  |  |  |  |
|  | No | | | 1.00 |  | | |  |  | 1.00 |  |  |
|  | Yes | | | 1.84 | 0.61-5.50 | | | 0.28 |  | 2.66 | 0.96-7.25 | 0.060 |
| **Work-related factors** | | | |  |  | | |  |  |  |  |  |
| *Grade* | | | | | | | |  |  |  |  |  |
|  | Officer | | | 1.00 | | |  |  |  | 1.00 |  |  |
|  | Soldier | | | 0.44 | | | 0.14-1.39 | 0.16 |  | 0.37 | 0.13-1.06 | 0.063 |
| *Military occupational function^c^* | | | | | | |  |  |  |  |  |  |
|  | Combat | | | 1.00 | | |  |  |  | 1.00 |  |  |
|  | CBC-Crew | | | 6.09 | | | 1.83-20.25 | 0.003 |  | 3.90 | 1.11-13.79 | 0.034 |
| *Total time working with similar tasks^d^* | | | | | | | |  |  |  |  |  |
|  | ≤ 24 months | | | 1.00 | | |  |  |  | 1.00 |  |  |
|  | ≥ 25 months | | | 2.87 | | | 0.89-9.29 | 0.078 |  | 2.39 | 0.75-7.63 | 0.142 |
| *Current Work ability, Physical^e^* | | | | | | | |  |  |  |  |  |
|  | High | | 1.00 | | |  | |  |  | 1.00 |  |  |
|  | Moderate | | 1.09 | | | 0.36-3.34 | | 0.88 |  | 1.18 | 0.43-3.29 | 0.745 |
| *Current Work ability, Mental^e^* | | | | | | | |  |  |  |  |  |
|  | High | | 1.00 | | |  | |  |  | 1.00 |  |  |
|  | Moderate | | 0.82 | | | 0.23-2.92 | | 0.76 |  | 1.06 | 0.34-3.29 | 0.921 |
| *Recovered when starting work* | | | | | | | |  |  |  |  |  |
|  | Always | | 1.00 | | |  | |  |  | 1.00 |  |  |
|  | Not always | | 1.08 | | | 0.25-4.63 | | 0.92 |  | 0.77 | 0.22-2.68 | 0.685 |
| *Sitting work* | | | | | | | |  |  |  |  |  |
|  | < 1/2 work day | 1.00 | | | |  | |  |  | 1.00 |  |  |
|  | ≥ 1/2 work day | 2.33 | | | | 0.68-8.01 | | 0.18 |  | 3.55 | 1.16-10.83 | 0.026 |
| *Computer work* | | | | | | | |  |  |  |  |  |
|  | < 1/4 work day | 1.00 | | | | *Reference* | |  |  | 1.00 | *Reference* |  |
|  | ≥ 1/4 work day | 2.87 | | | | 0.89-9.29 | | 0.078 |  | 3.12 | 1.04-9.31 | 0.042 |
| *Vibrating floor/seat* | |  | | | |  | |  |  |  |  |  |
|  | < 1/4 work day | 1.00 | | | | *Reference* | |  |  | 1.00 | *Reference* |  |
|  | ≥ 1/4 work day | 2.02 | | | | 0.67-6.09 | | 0.210 |  | 2.23 | 0.78-6.37 | 0.135 |

*^a^* Odds ratio based on penalized likelihood (Firth) binary logistic regressions

*^b^*Pain, within 6 month prior to baseline.

^c^MOS dichotomized to *Combat* ( Infantry /Ranger) and *combat craft* (*CBC)-crew* in order to retain statistical power for this anlyisis.

*^d^Total time working with similar tasks* dichotomized to *≤ 24 months* (*< 12 and 12-24 months merged)*  and *≥ 25 months* in order to retain statistical power for this analysis.

*^e^*Current Work ability in regards to demands of work

Table S3. Multiple regression analyses: final unadjusted and adjusted ^a^ odds ratio (OR) ^b^ for back pain limiting work ability within six months from baseline.

|  | |  | Unadjusted | | | |  | Adjusted^a^ | | |
| --- | --- | --- | --- | --- | --- | --- | --- | --- | --- | --- |
|  | |  | OR^b^ | | 95% CI | p-value |  | OR^b^ | 95% CI | p-value |
| *Body height (m)* | | | | |  |  |  |  |  |  |
|  | | < 1.86 | | 1.00 | *Reference* |  |  | 1.00 | *Reference* |  |
|  | | ≥ 1.86 | | 4.99 | 1.53-16.25 | 0.008 |  | 4.30 | 1.31-14.13 | 0.016 |
| *Military occupation specialty^c^* | | | | | |  |  |  |  |  |
|  | Combat | | | 1.00 | *Reference* |  |  | 1.00 | *Reference* |  |
|  | CBC-Crew | | | 6.33 | 1.74-23.00 | 0.005 |  | 5.87 | 1.58-21.81 | 0.008 |

^a^Adjusted for back pain within six months of baseline.

*^b^*Odds ratio based on penalized likelihood (Firth) binary logistic regressions

^c^MOS dichotomized to *Combat* ( Infantry /Ranger) and *combat craft* (*CBC)-crew* in order to retain statistical power for this analysis.

Table S4. Multiple regression analyses: final odds ratio (OR)^a^ for back pain limiting work ability within 12 months from baseline.

|  | |  | OR^a^ | | 95% CI | p-value |  |
| --- | --- | --- | --- | --- | --- | --- | --- |
| *Body height (m)* | | | | |  |  |  |
|  | | < 1.86 | | 1.00 | *Reference* |  |  |
|  | | ≥ 1.86 | | 4.55 | 1.53-13.57 | 0.007 |  |
| *Prior back pain^d^* | | | | | |  |  |
|  | no | | | 1.00 | *Reference* |  |  |
|  | yes | | | 6.64 | 1.78-24.78 | 0.005 |  |

*^a^*Odds ratio based on penalized likelihood (Firth) binary logistic regressions

^b^Back pain, within 6 month prior to baseline.
